# Data on point cloud scanning and ground radar of composite lining in jointly constructed tunnel

**DOI:** 10.1016/j.dib.2022.107993

**Published:** 2022-02-25

**Authors:** Jia-Xuan Zhang, Ning Zhang, Ye-Shuang Xu

**Affiliations:** a Department of Civil Engineering, School of Naval Architecture, Ocean, and Civil Engineering, Shanghai Jiao Tong University, Shanghai 200240, China; b Department of Civil and Environmental Engineering, College of Engineering, Shantou University, Shantou, Guangdong 515063, China; c Shanghai Key Laboratory for Digital Maintenance of Buildings and Infrastructure, Shanghai Jiao Tong University, Shanghai 200240, China

**Keywords:** Jointly constructed tunnel, Terrestrial laser scanning, Over-under-excavation, Ground-penetrating radar, Heterogeneity

## Abstract

The present dataset pertains to field records of construction quality of composite lining in a jointly constructed tunnel. The dataset includes the original mining surface profile data collected by the terrestrial laser scanning (TLS) and radar information on backfill quality outside the segmental lining which was obtained by the ground-penetration radar (GPR) detection. The point cloud data of the mining surface was further processed and compared with the design tunnel model to evaluate the level of over and under- excavation. The radargram provides details on the variation of the signal waveform by which the heterogeneity of backfill can be recognized. The dataset can be used to verify that the voids are prone to occur in the outside backfill of the composite lining. Furthermore, this dataset provides a method for detecting and preventing the defects of the composite lining and also facilitates the post-construction treatment. Additional foreseeable use of this dataset includes providing modeling material for researchers interested in knowing how voids in backfill influence the behavior of composite lining. As a supplement, this dataset supports the numerical analysis outlined in the article titled “Numerical evaluation of segmental tunnel lining with voids in outside backfill” [1].

## Specifications Table

**Table:** 

Subject	Civil and Structural Engineering
Specific subject area	Geotechnical engineering and engineering geology
Type of data	Figure, table and text
How data were acquired	Terrestrial laser scanning inspection, ground-penetrating radar detection and calculation
Data format	Raw and analysed
Description of data collection	The point cloud of the tunnel was collected from the terrestrial laser scanning (TLS) inspection. The under/over-excavation were calculated by comparing the point cloud with the design tunnel model. The radargram was derived from the ground-penetrating radar (GPR) detection.
Data source location	Xuexiang Station-Gankeng Station, Metro Line No. 10, Shenzhen, China.
Data accessibility	The relevant raw data can be found in the supplement file (Point cloud.zip, Radagrams.docx, and Central axis.xlsx). Repository name: Mendeley Data Data identification number: 10.17632/c9mmbmkwcj.2 Direct URL to data: https://data.mendeley.com/datasets/c9mmbmkwcj/2
Related research article	This article is submitted as companion paper of: J.X. Zhang, N. Zhang, A. Zhou, S.L. Shen, Numerical evaluation of segmental tunnel lining with voids in outside backfill, Undergr. Space, in press. https://doi.org/10.1016/j.undsp.2021.12.007.

## Value of the Data

• The dataset contains the point cloud of tunnel surface profile, which facilitates the analysis of over and under-excavation in mining method tunnels.
• The dataset provides radar information about the tunnel and can be used to determine the location of heterogeneity behind the segmental lining.
• The collection process of the dataset provides a method to timely find the construction defect, such as the leakage of groundwater [1], [2], [3], ground settlement [4] and structure deformation [5], especially to facilitates the treatment when the composite lining is constructed.
• This dataset can be conveniently reused by other researchers as an additional part of a larger engineering dataset. The point cloud dataset including the geometry information is of benefit to the parameter optimization [6], [7], [8], risk assessment [9], [10], [11] in the construction of tunnels.

## 1 Data Description

**Fig. 1:**
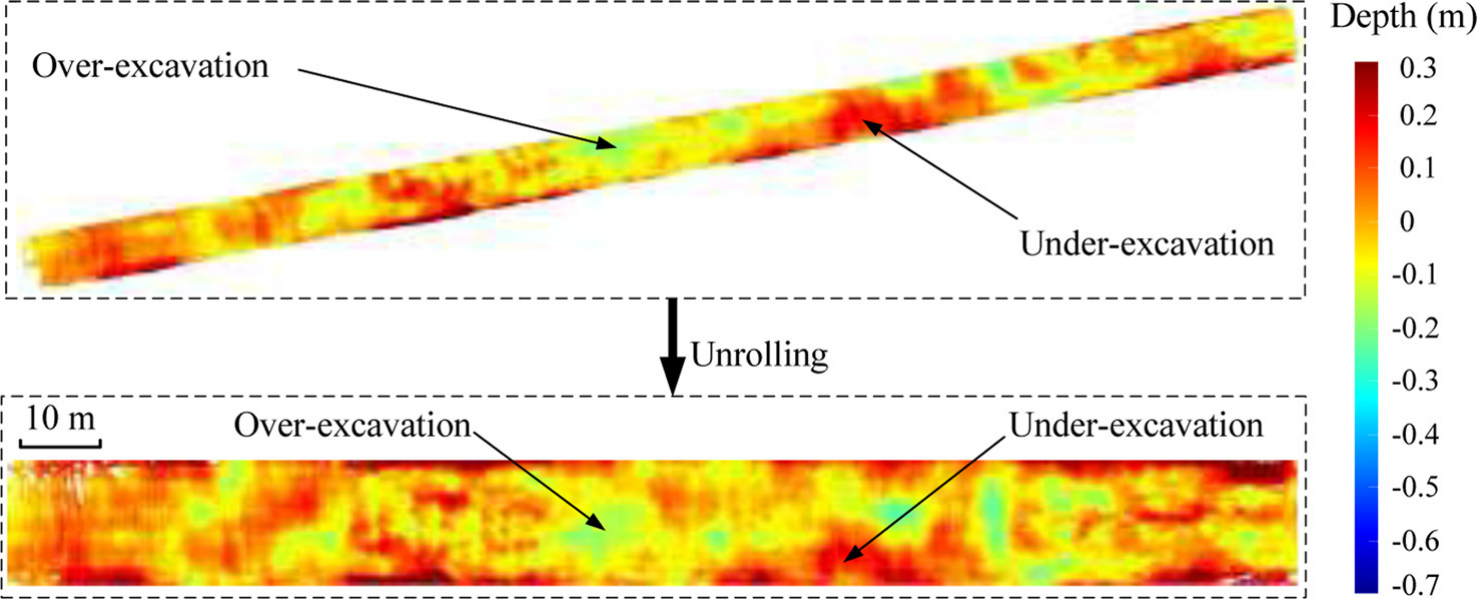
Over-under-excavation contour map of the tunnel.

**Fig. 2:**
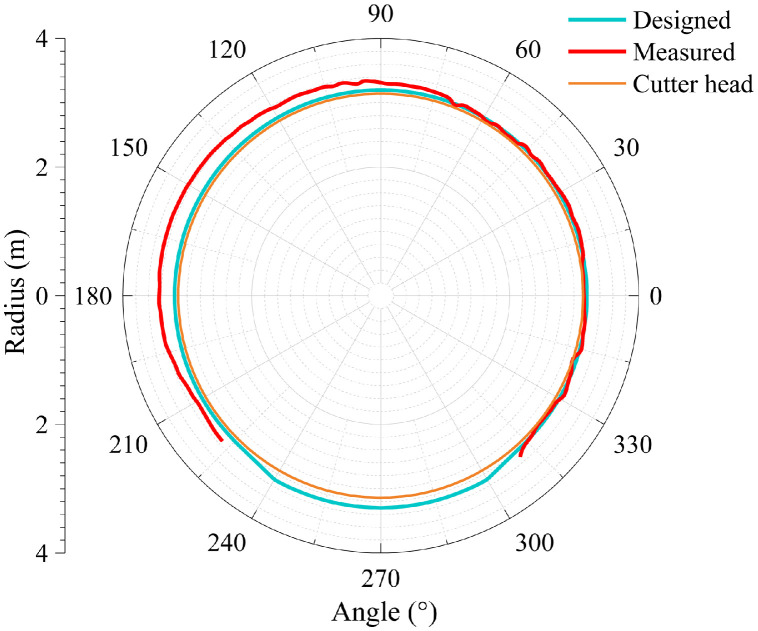
Section at distance of 30 m.

**Fig. 3:**
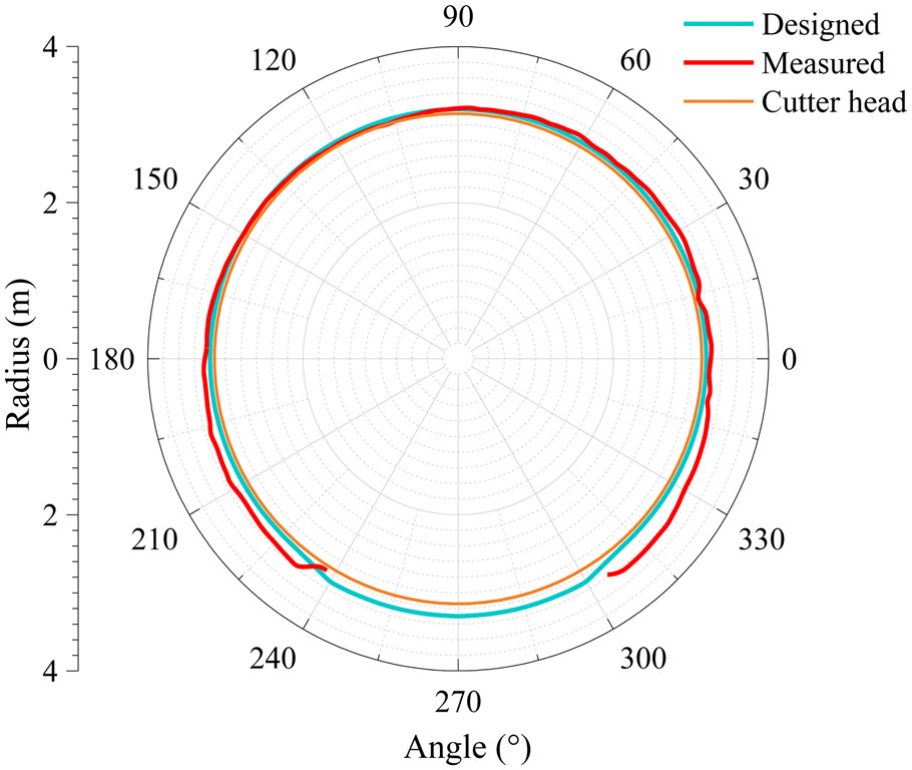
Section at distance of 75 m.

**Fig. 4:**
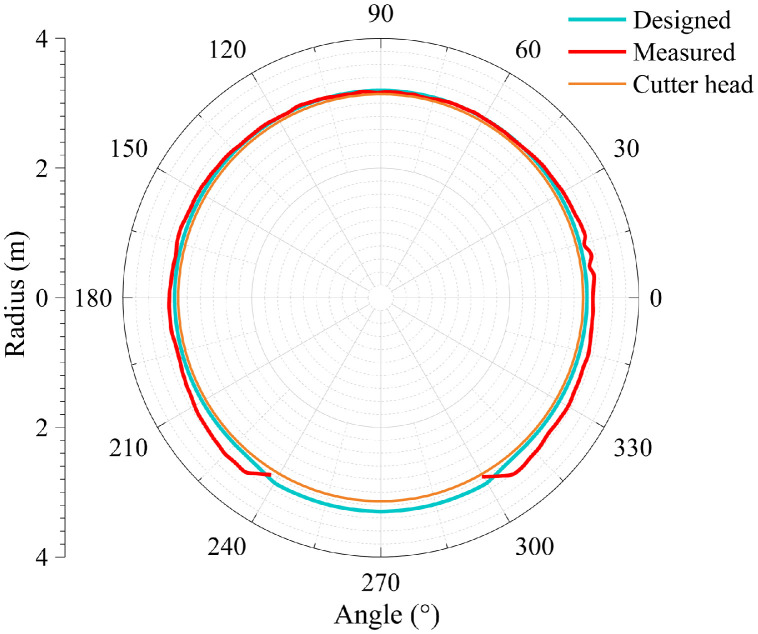
Section at distance of 80 m.

**Fig. 5:**
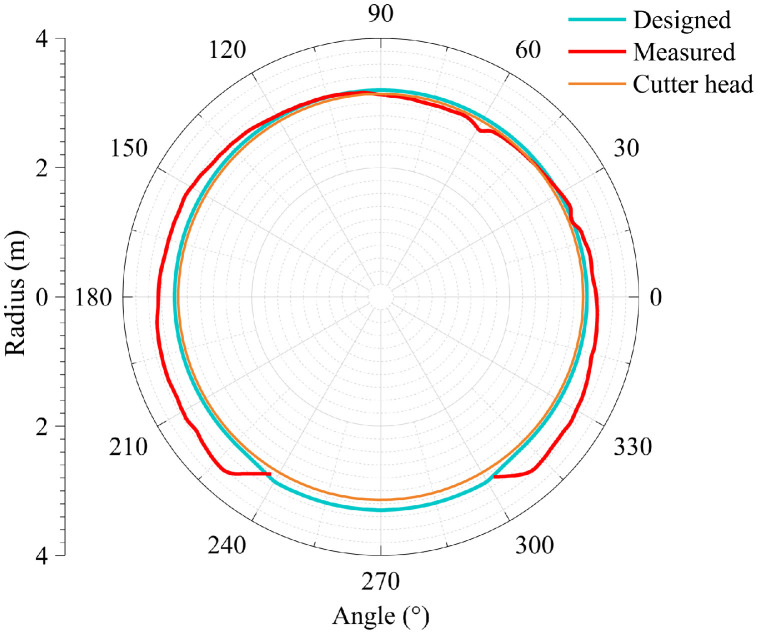
Section at distance of 115 m.

**Fig. 6:**
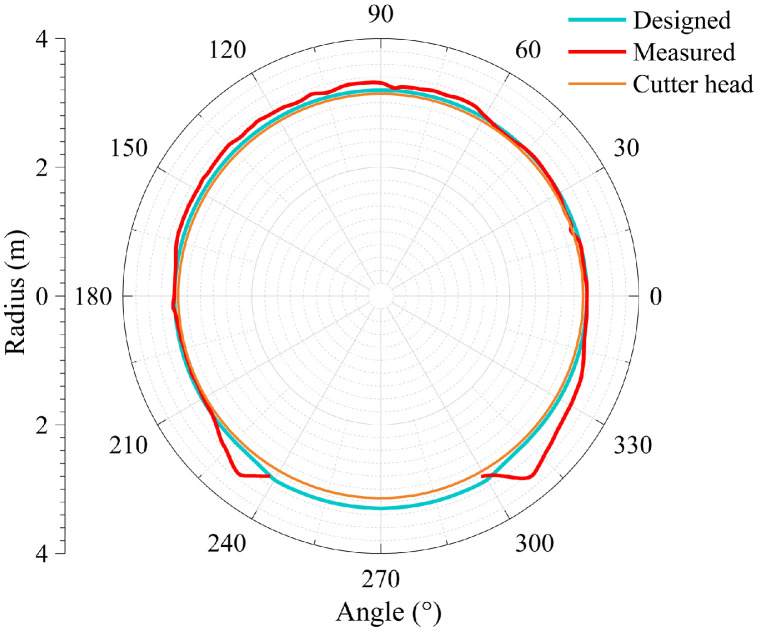
Section at distance of 135 m.

**Fig. 7:**
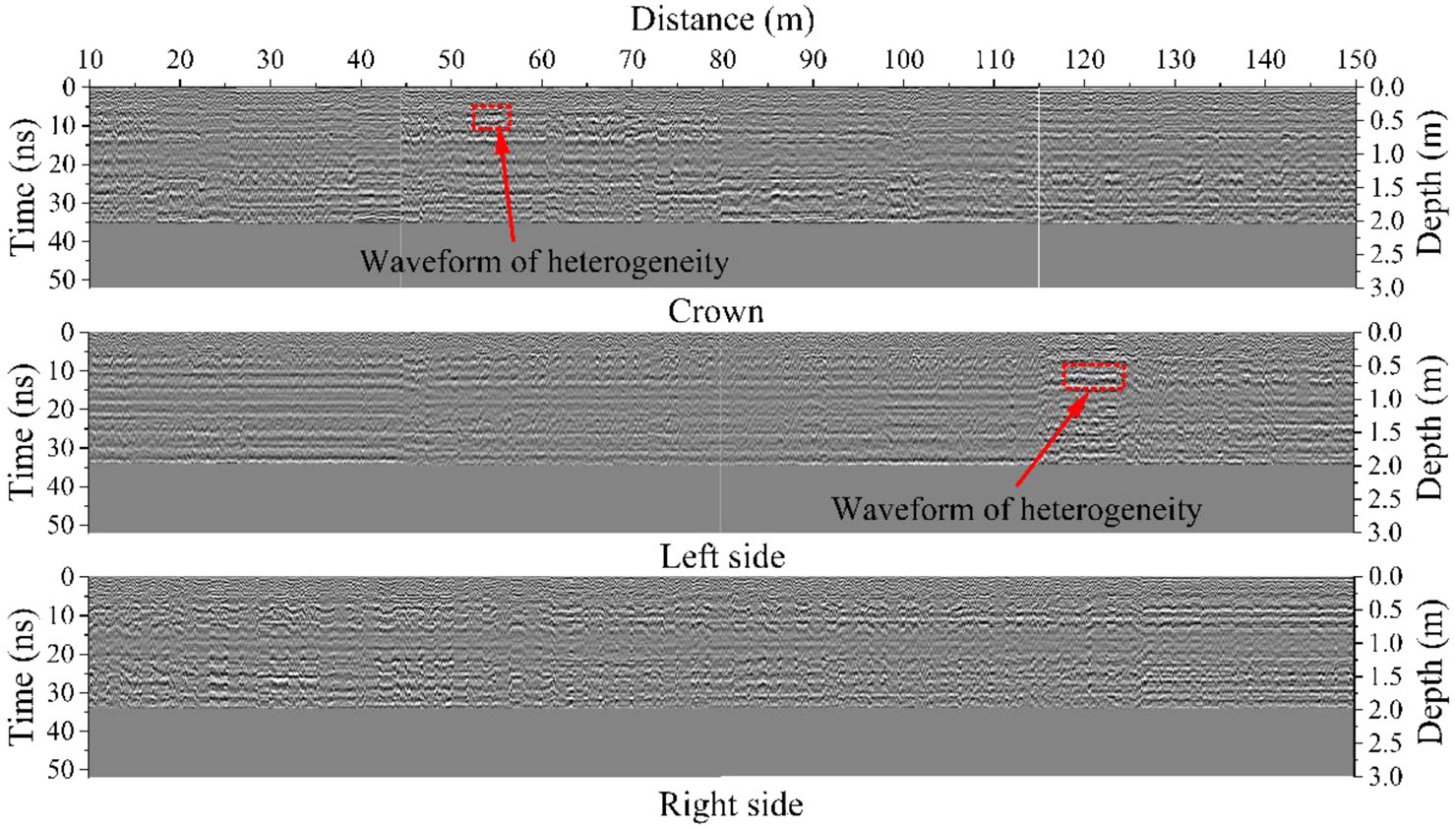
Radargram in distance range of 10–150 m.

The dataset in this article was collected in the tunnel (referred to as the Xue-gan tunnel) connecting Xuexiang and Gankeng stations of Shenzhen Metro Line 10 [1]. It consists of the point cloud (Point cloud.zip) obtained by the terrestrial laser scanning (TLS), the designed central axis (central axis.xlsx) and the radagrams (Radagrams.docx) collected by ground-penetration radar (GPR) detection [12]. All of the data was processed based on the conjunction with the study and the construction project. The point cloud provides the information of Cartesian coordinates (*x, y, z*) and intensity of each point. The designed central axis is denoted by the central point Cartesian coordinates (*x, y, z*) of some selected profiles of the tunnel. The radagrams display the signal waveforms of GPR detection at the locations of crown, left and right side of the tunnel along the alignment from 10 to 150 m. Based on the data, the over/under-excavation and heterogeneity of the tunnel were analysed. The over/under-excavation of the entire tunnel is displayed in a contour map (see Fig. 1) derived from the difference between the point cloud and design model of the tunnel. Generally, the over/under-excavation information is a crucial factor of the deformation-related issues in tunnels [13], [14], [15]. Several transverse sections were generated to compare the dimensions of the measured, design and cutter head profile Table 1. lists the over and under-excavation ratio and maximum values of the over/under-excavation. The rows of Table 1 correspond to the 34 transverse sections between distance of 0–165 m at intervals of 5 m. The over and under-excavation ratio is defined by calculating the proportion of the over/under-excavation range in the circumference direction. From the statistics in the table, the amount of over-excavation of the tunnel is more than that of under-excavation Fig. 2, Fig. 3, Fig. 4, Fig. 5, Fig. 6. show representative sections where tunnel was seriously under-excavated Fig. 7. presents the radargrams collected by the ground-penetration radar (GPR), and the heterogeneity behind the segmental lining was marked. In the radargrams, the left and right vertical axis denotes the time that an electromagnetic wave is emitted to receive and the depth from the concrete segment surface, respectively. The horizontal axis denotes the distance from the starting point of tunnel.

**Table 1:** Statistics of over-under excavation.

No.	*D*	*ω*_1_	*ω*_2_	*M*_1_	*M*_2_
1	0	61.8	1.3	22	12
2	5	100.0	-	25	-
3	10	100.0	-	39	-
4	15	100.0	-	53	-
5	20	100.0	-	44	-
6	25	76.7	4.0	46	1
7	30	60.0	37.0	26	7
8	35	100.0	-	27	-
9	40	91.7	3.3	30	8
10	45	100.0	-	50	-
11	50	100.0	-	64	-
12	55	100.0	-	69	-
13	60	93.3	-	54	-
14	65	95.0	-	56	-
15	70	73.3	5.0	55	3
16	75	38.33	20.7	25	7
17	80	45.0	20.0	22	4
18	85	85.0	-	21	-
19	90	90.0	1.3	22	10
20	95	88.3	-	37	-
21	100	75.0	0.7	38	3
22	105	85.0	-	39	-
23	110	81.7	-	33	-
24	115	65.0	25.3	43	22
25	120	93.3	0.7	40	6
26	125	70.0	1.3	30	2
27	130	76.7	-	34	-
28	135	60.0	27.2	46	11
29	140	72.5	11.8	46	8
30	145	95.0	-	44	-
31	150	100.0	-	58	-
32	155	93.3	-	64	-
33	160	67.5	7.3	70	6
34	165	95.0	-	46	-

*Note: D* = Distance from the starting point of jointly constructed tunnel; *ω*_1_ = Over-excavation ratio (%); *ω*_2_ = Under-excavation ratio (%); *M*_1_ = Maximum over-excavation (cm); *M*_2_ = Maximum under-excavation (cm).

## 2 Experimental Design, Materials and Methods

**Fig. 8:**
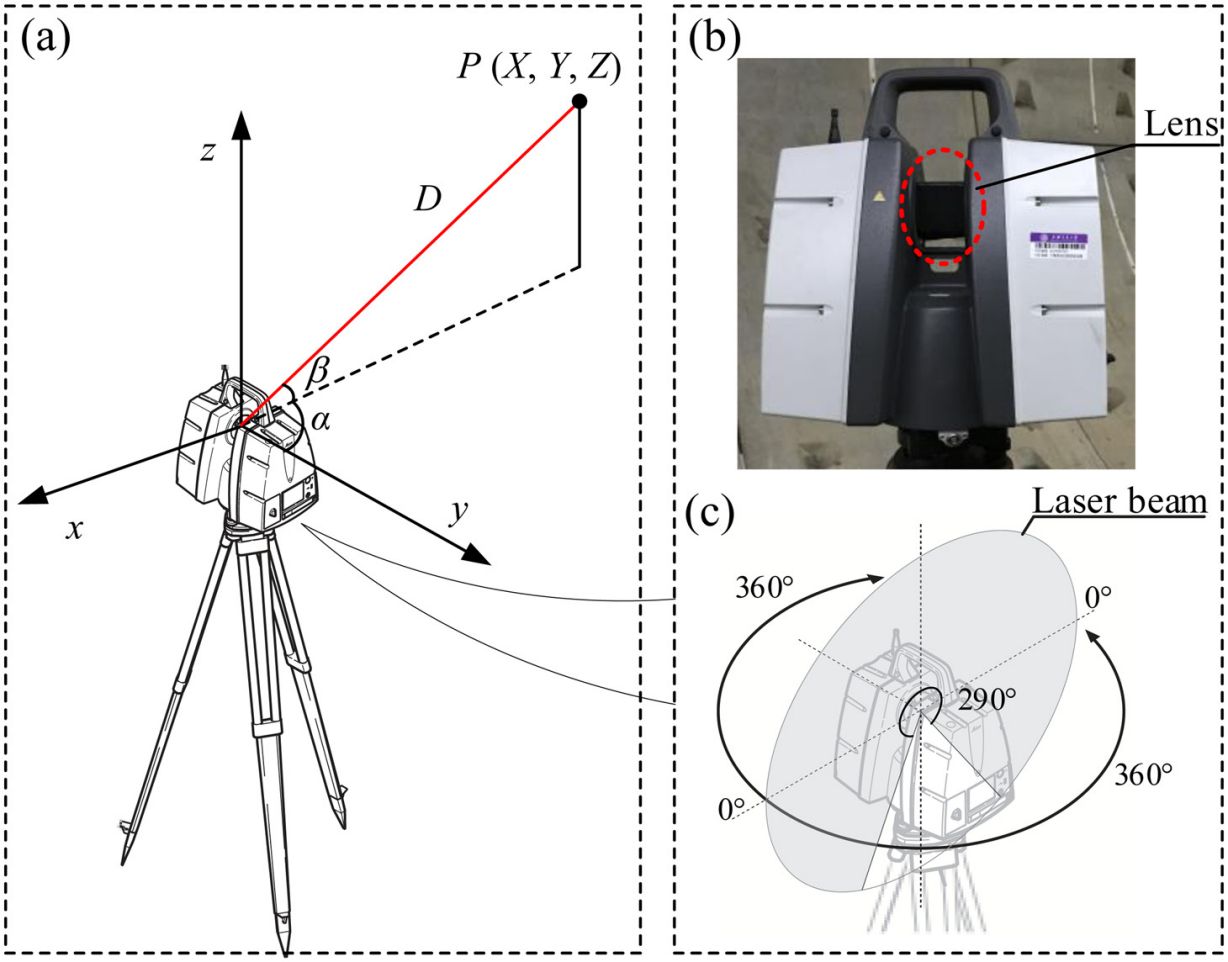
Schematic diagram of the TLS instrument: (a) stand view of the device, (b) picture of main device, (c) laser cloud (recreated based on [16]).

The point cloud data was acquired by using the terrestrial laser scanning (TLS). The TLS instrument was Leica ScanStation P40, of which the specifications are listed in Table 2 and the schematic diagram is shown in Fig. 8 [16]. Leica Scan Station P40 was used to indirectly obtain the distance to the object by the time difference between the transmitted and received laser pulse signals. During the scanning process, the laser scanner rotates horizontally at a slow and constant speed from 0° to 360° while the lens rotates at a high speed vertically. The laser transmitter emits a laser pulse to the target through the lens and records the return time *t* after reflected by the target *P* (point on the tunnel in this study). Therefore, the distance *D* from the Leica Scan Station and the target was calculated by D=ct/2, where *c* is the speed of light. The relative coordinates (*X, Y, Z*) of target *P* can be expressed as below:

$$\left\{ \begin{array}{c} X=D\cdot \cos\alpha \cdot \cos\beta \\ Y=D\cdot \cos\beta \cdot \sin\alpha \\ Z=D\cdot \sin\beta \end{array}\right.$$

where *α* is the horizontal angle between *P* and *y*-axis, and *β* is the vertical angle of target *P*.

**Table 2:** Specifications of Leica ScanStation P40.

Measurement type	Time-of-flight
Wavelength	1550 nm (invisible) / 658 nm (visible)
Beam divergence	0.23 mrad (FWHM, full angle)
Range accuracy	1.2 mm + 10 ppm over full range
Accuracy 3D position	3 mm at 50 m; 6 mm at 100m
Angular accuracy	8″ horizontal; 8″ vertical
Beam diameter at exit	5.8 mm
Beam diameter at 10 m	8.1 mm
Beam diameter at 20 m	3.5 mm
Maximum range	up to 270 m at 34%; 180 m at 18%; 120 m at 8% reflectivity
Scan rate	up to 1000,000 points/ second

In the entire tunnel, 6 stations were installed to inspect the inner surface of the tunnel, the resolution of the laser scanner was set to 3 mm at a distance of 10 m. Between two adjacent stations, three black-white targets were mounted on the different locations of the tunnel profile, and the coordinates of each target were measured by the total station. When the measurement was finished, the recorded data obtained from each station were registered and calibrated based on the coordinates of the black-white targets, so that the coordinates of the whole point cloud can be produced. The point cloud data consists of more than 150 million points, and the information of each point includes the coordinates and intensity. To obtain the contour map, the Cartesian coordinates were transformed to the cylinder coordinates. The algorithm of the contour map generation can be seen in Table 4. The sound of reflection was recorded by using the GSSI SIR 3000 ground-penetration radar detection in field and then simulated to signal waves. The specifications of GSSI SIR 3000 GPR can be seen in Table 3 [17]. Along the alignment of the tunnel, five detection line were uniformly fixed at the upper half profile. The obtained signal waves need to be further processed including denoising, amplitude enlargement and outliers processing. Comparing the signal waves obtained at the same depth, the location of the heterogeneity behind the segmental lining can be recognized where both the phase and amplitude have changed significantly. These data would provide assistance to the assessment of potential risks during the construction and operation of tunnels [18], [19], [20].

**Table 3:** Specifications of GSSI SIR 3000.

Scan rate examples	220 scans/s at 256 samples/s, 16 bit 120 scans/s at 512 samples
Number of samples per scan	256, 512, 1024, 2048, 4096, 8192
Transmit rate	Up to 100 KHz
Time range	0–8000 ns full scale, user-selectable Gain: Manual or automatic, 1–5 gain points (−20 to +80 dB)
Filters	Vertical: Low pass and high pass IIR and FIR Horizontal: Stacking, background removal

**Table 4:** Generation process of over-under-excavation contour map.

**Algorithm 1**: The generation process of over-under-excavation contour map [21].			
**Input**: dataset $\left\{P_{i}=\left(x_{i},y_{i},z_{i}\right),i=1,...,n\right\}$, where *n* is the point number of point cloud *P*, (x,y,z) are Cartesian coordinates of point $P_{i}$.			
**1:** Transform the measuring coordinate system (O−xyz) to the local coordinate system ($O^{\prime }-x^{\prime }y^{\prime }z^{\prime }$)			
(i) Compute the rotation matrix ***A***:			
$A=\left[\begin{array}{ccc} \cos\alpha_{1} & \cos\alpha_{2} & \cos\alpha_{3}\\ \cos\beta_{1} & \cos\beta_{2} & \cos\beta_{3}\\ \cos\gamma_{1} & \cos\gamma_{2} & \cos\gamma_{3} \end{array}\right]$			
where α,β,γ are the angles between the axis of O−xyz and$O^{\prime }-x^{\prime }y^{\prime }z^{\prime }$, which are listed as below:			

Axis	*x*	*y*	*z*

$x^{\prime }$	$\alpha_{1}$	$\beta_{1}$	$\gamma_{1}$
$y^{\prime }$	$\alpha_{2}$	$\beta_{2}$	$\gamma_{2}$
$z^{\prime }$	$\alpha_{3}$	$\beta_{3}$	$\gamma_{3}$

(ii) Compute the coordinate values of points in $O^{\prime }-x^{\prime }y^{\prime }z^{\prime }$			
$\left[x^{\prime }\;y^{\prime }\;z^{\prime }\right]=\left[x-x_{0}\;y-y_{0}\;z-z_{0}\right]A$			
where $\left(x^{\prime },y^{\prime },z^{\prime }\right)$ are the coordinates in $O^{\prime }-x^{\prime }y^{\prime }z^{\prime }$, (x,y,z) are the coordinates in O−xyz, $\left(x_{0},y_{0},z_{0}\right)$ are the coordinates of $O^{\prime }$			
2: Transform Cartesian coordinates to Cylindrical coordinates			
(i) Compute the Euclidean distance ρ from each point to $z^{\prime }$-axis: $\rho =\sqrt{x{}^{\prime 2}+y{}^{\prime 2}}$			
(ii) Compute the azimuth angle φ between the reference direction on the $x^{\prime }-O-y^{\prime }$ plane and the line from the origin to the projection of each point on the plane:			
$\varphi =\left\{ \begin{array}{c} 0x^{\prime }=0,y^{\prime }=0\\ \arcsin\left(\frac{y^{\prime }}{\rho }\right)x^{\prime }\geq 0\\ \arctan\left(\frac{y^{\prime }}{x}\right)x^{\prime }>0\\ -\arcsin\left(\frac{y^{\prime }}{\rho }\right)+\pi x^{\prime }<0 \end{array}\right.$			
(iii) Assign the Cylindrical coordinates values of each point ${P_{i}}^{\prime }$_:_${P_{i}}^{\prime }=\left(\rho ,\varphi ,z^{\prime }\right),i=1,2,3\ldots n$			
3: Compute the difference *d* between the measured and designed model			
d=ρ−R, where *R* is the designed radius of the tunnel			
4: Generate the over-under-excavation contour map			
(i) Unroll the measured point cloud along the central axis: $U_{i}=\left(z^{\prime },\varphi ,d\right)$,where $U_{i}$ is the point of unrolled point cloud *U*.			
(ii) Render the colour of point cloud *U* according to $z^{\prime }$ values			
**Output**: the over-under-excavation contour map.			

## Ethics Statements

The present work did not involve the use of human subjects, animal experiments, or data collected from social media platforms.

## CRediT authorship contribution statement

**Jia-Xuan Zhang:** Methodology, Software, Writing – original draft. **Ning Zhang:** Conceptualization, Validation, Writing – review & editing. **Ye-Shuang Xu:** Supervision, Writing – review & editing.

## Declaration of Competing Interest

The authors declare that they have no known competing financial interests or personal relationships that could have appeared to influence the work reported in this paper.
